# Synergy of circulating miR-212 with markers for cardiovascular risks to enhance estimation of atherosclerosis presence

**DOI:** 10.1371/journal.pone.0177809

**Published:** 2017-05-30

**Authors:** Hye Seon Jeong, Jee-Yeon Kim, Seo Hyun Lee, Junha Hwang, Jong Wook Shin, Kyu Sang Song, Sukhoon Lee, Jei Kim

**Affiliations:** 1 Neuroepigenetics Laboratory, School of Medicine and Hospital, Chungnam National University, Daejeon, South Korea; 2 Department of Neurology, School of Medicine and Hospital, Chungnam National University, Daejeon, South Korea; 3 Regional Cerebrovascular Center, Chungnam National University Hospital, Daejeon, South Korea; 4 Department of Information and Statistics, College of Natural Science, Chungnam National University, Daejeon, South Korea; 5 Department of Pathology and Hunan Bio-Resource Bank, School of Medicine and Hospital, Chungnam National University, Daejeon, South Korea; University of Kansas Medical Center, UNITED STATES

## Abstract

Synergy of specific microRNAs (miRNAs) with cardiovascular risk factors to estimate atherosclerosis presence in ischemic stroke patients has not been investigated. The present study aimed to identify atherosclerosis-related circulating miRNAs and to evaluate interaction with other cardiovascular markers to improve the estimation of atherosclerosis presence. We performed a miRNA profiling study using serum of 15 patients with acute ischemic stroke who were classified by the presence of no (n = 8) or severe (n = 7) stenosis on intracranial and extracranial vessels, which identified miR-212, -372, -454, and -744 as miRNAs related with atherosclerosis presence. Of the 4 miRNAs, only miR-212 showed a significant increase in expression in atherosclerosis patients in a validation study (atherosclerotic patients, n = 32, non-atherosclerotic patients, n = 33). Hemoglobin A1c, a high-density lipoprotein cholesterol, and lipoprotein(a), both established risk markers, were independently related with atherosclerosis presence in the validation population. miR-212 enhanced the accuracy of atherosclerosis presence by the three existing markers (three markers, 78.5%; three markers+miR-212, 84.6%, *P*<0.05) and significantly added to the area under the receiver operating characteristic curve (three markers, 0.8258; three markers+miR-212, 0.8646, *P*<0.05). The inclusion of miR-212 increased the reclassification index calculated using net reclassification improvement (0.4527, *P*<0.05) and integrated discrimination improvement (0.0737, *P*<0.05). We identified circulating miR-212 as a novel marker of atherosclerosis. miR-212 enhanced the estimation of atherosclerosis presence in combination with hemoglobin A1c, high-density lipoprotein cholesterol, and lipoprotein(a). Thus, miR-212 is expected to improve the estimation of atherosclerosis using peripheral blood of patients.

## Introduction

Atherosclerosis is a leading cause of cardiovascular diseases such as stroke and coronary artery disease.[1] The likelihood of developing of atherosclerosis and cardiovascular disease varies individually.[2] To predict the likelihood of atherosclerosis development, standard (age, sex, and race) and established (hypertension, diabetes, hyperlipidemia, obesity, smoking, and alcohol drinking, etc.) risk factors have been used.[2,3] However, the burden of morbidity and mortality by cardiovascular diseases remains high.[2] Thus, there is a pressing need for novel atherosclerosis markers to improve the predictability of the traditional standard and established risk factors.[4] Recently, C-reactive protein[5,6] and multiple genetic polymorphisms[7] have been shown to improve the prediction of cardiovascular diseases when used in combination with the traditional risk factors. However, new markers are still needed to explain environmental influences on the development of atherosclerosis and cardiovascular diseases.[8]

Several microRNAs (miRNAs) in the circulation recently emerged as possible biomarkers mirroring environmental influences such as inflammation and mechanosensitivity related to atherosclerosis.[9–11] While these studies usually showed a possible relationship between the specific miRNAs and atherosclerosis, the accuracy and increment in discriminatory power for atherosclerosis prediction were generally not validated.[9] In particular, synergistic effects between miRNAs and standard and established risk markers have not been well studied. Moreover, different studies obtained inconsistent results regarding the relationships between miRNAs and atherosclerosis, which could be explained by different inclusion criteria for age, timing of sampling, disease classification, and disease severity.[9]

The present study aimed to identify and validate specific miRNAs related with atherosclerosis presence in the serum of atherosclerosis patients and to evaluate whether the validated miRNAs enhance the estimation power of the standard and established risk factors. For this purpose, the study was outlined based on a previous protocol for the evaluation of novel markers:[3] 1) identification and validation of miRNAs in a population of ischemic stroke patients stratified by age, acute ischemic lesion size, and severity of atherosclerosis, 2) evaluation of individual and combinational significances of the standard and established risks as well as the identified miRNAs to estimate atherosclerosis presence in the stratified patients, and, 3) evaluation of the accuracy and discriminatory power of miRNAs to enhance estimation of atherosclerosis presence.

## Methods

### Human subjects and cardiovascular risk factor evaluations

**Fig 1:**
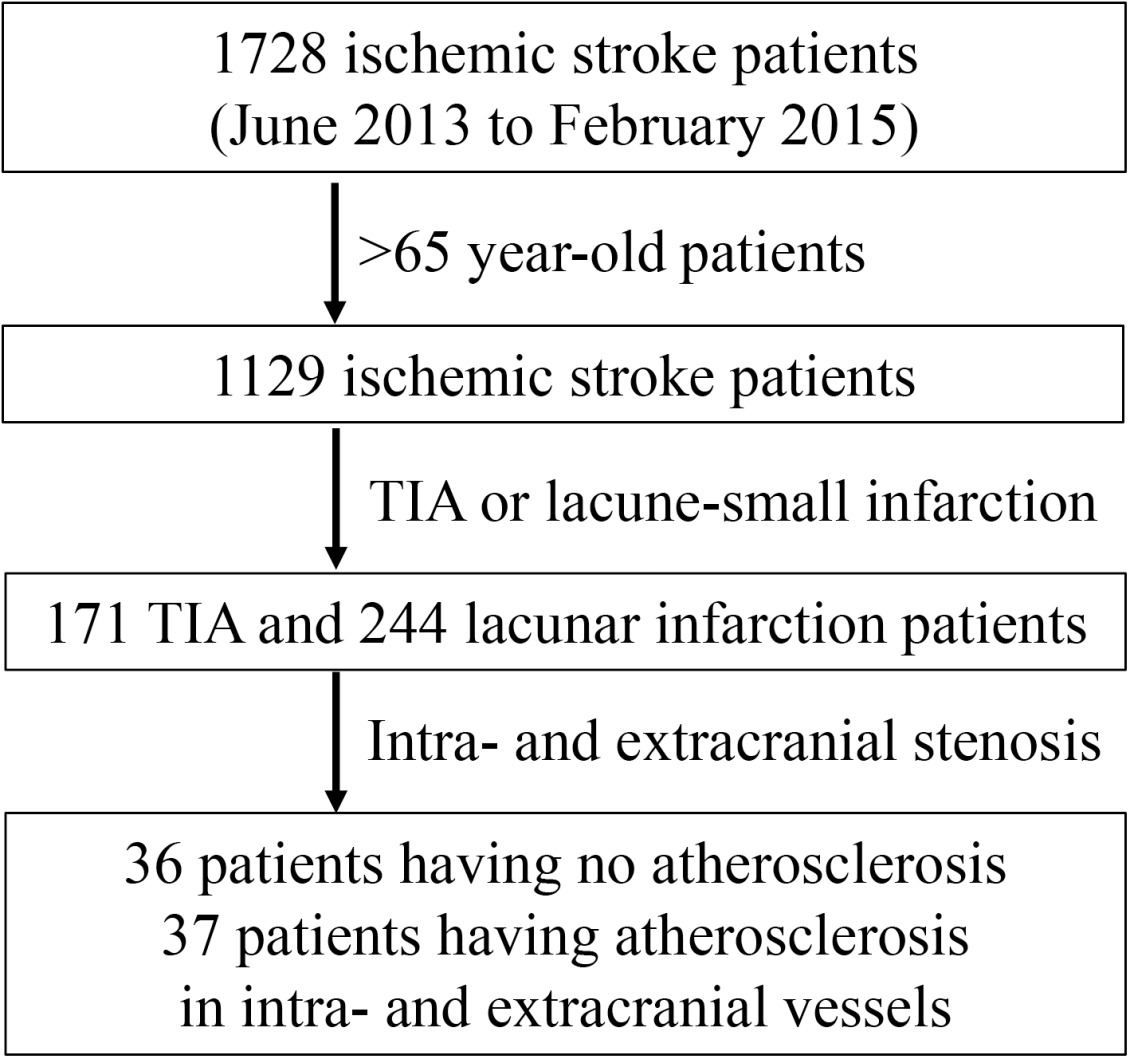
Flow diagram of patient enrollment from the Human Bio-Resource Bank of Chungnam National University Hospital.

For the present study, we selected patients from 1728 ischemic stroke patients whose serum was prospectively stored in the Human Bio-Resource Bank of a University Hospital between June 2013 and February 2015. First, we included patients >65 years of age (1129 patients) from the total patients having samples deposited in the Human Bio-Resource Bank. From these, 171 transient ischemic attack (TIA) patients, who showed transient neurological deficit and no abnormal signal intensity on diffusion-weighted magnetic resonance images (DWI) checked within 24 h after symptom onset, and 244 lacunar or small infarction patients, who showed focal neurologic deficit and <2.0-cm-high signal on the initial DWI, were selected. Finally, 36 atherosclerotic patients, who had severe stenosis in the intracranial and extracranial vessels, and 37 non-atherosclerotic patients, who had no stenosis in the evaluated vessels, were enrolled in the present study (Fig 1).

TIA, transient ischemic attack, transient neurological deficits lasting <24 h and no acute lesion on diffusion weighted magnetic resonance image (DWI); lacune-small infarction, neurological deficits lasting >24 h and <2.0 cm acute lesion on DWI; intracranial atherosclerosis, >3 vessels having >50% of stenosis on 11 intracranial vessels (middle cerebral arteries, anterior cerebral arteries, posterior cerebral arteries, intracranial internal carotid arteries, vertebral arteries of both sides, and basilar artery) on time of flight-magnetic resonance angiography; extracranial atherosclerosis, >2 vessels having >50% stenotic area on carotid duplex sonography.

Stenosis status of intra- and extracranial vessels was evaluated at admission using time-of-flight magnetic resonance angiography (MRA) and carotid duplex ultrasonography. Intracranial stenosis was measured in 11 intracranial vessels (internal carotid arteries, middle cerebral arteries, anterior cerebral arteries, posterior cerebral arteries, vertebral arteries of both sides, and basilar artery) on MRA.[10] Extracranial stenosis was measured in 6 extracranial vessels (common carotid artery, internal carotid arteries, and external carotid artery of both sides) on carotid duplex ultrasonography. The criteria to determine severe stenosis in the present study were: intracranial stenosis in >3 intracranial vessels having >50% of stenosis on MRA and simultaneous extracranial stenosis in >2 extracranial vessels having >50% stenotic area on carotid duplex ultrasonography.

The presence of previous ischemic brain lesion was also reviewed in initial magnetic resonance imaging (MRI). The previous lesions were defined as subacute (high on DWI, low on apparent diffusion coefficient [ADC], low on T1-weighed [T1WI], high on T2-weighted [T2WI], and high signal intensity on fluid-attenuated inversion recovery [FLAIR] images), and chronic cerebral infarction (low on DWI, high on ADC, low on T1WI, high on T2WI, and low signal intensity on FLAIR images).[12] And, the previous lesions were classified into lacunar (<2cm) and branch infarction.

All patients included were also evaluated National Institute of Health Stroke Scale (NIHSS) checked at emergency room (ER) and discharge, the presence of standard and established cardiovascular risk factors, including age, sex, and history of medication for hypertension and diabetes, smoking, and alcohol consumption. (S1 Table) Markers of established risk factors, including body mass index for obesity,[11,13] hemoglobin A1c (HbA1c)[14–16] and fasting blood glucose[17] for diabetes, total cholesterol, triglyceride, low-density lipoprotein cholesterol (LDL-C), high-density lipoprotein cholesterol (HDL-C), lipoprotein(a), and apolipoprotein-A and -B for lipid metabolism,[18] and high-sensitivity C-reactive protein (hs-CRP)[19] and homocysteine[20,21] for inflammation and endothelial dysfunction, were checked in fasting state within 24 h after admission (Table 1, S1 Table).

**Table 1:** Characteristics of the clinical and laboratory cardiovascular risk factors in included patients.

		Non-atherosclerotic (mean ± SD) (n = 33)	Atherosclerotic (mean ± SD) (n = 32)	*p*-value
Demographics	Sex (men:women)	19:14	19:13	1.000
	Age (years)	69.3 ±4.9	71.4 ± 5.8	0.110
	Height (cm)	161.2 ± 9.3	160.2 ± 9.1	0.680
	Weight (kg)	62.8 ± 9.7	62.1 ± 10.2	0.773
	Body mass index	24.1 ± 2.2	24.1 ± 3.4	0.907
Risk factor history	Hypertension	15:18 (45.5%)	24:8 (75%)	0.023
	Diabetes	5:28 (15.2%)	18:14 (56.3%)	0.001
	Smoking	8:25 (24.2%)	6:26 (18.8%)	0.764
	Alcohol	3:30 (9.1%)	3:29 (9.4%)	1.000
NIHSS	At ER	1.3±1.7	1.1±1.7	0.542
	At discharge	0.7±1.3	0.8±1.3	0.779
Diagnosis	TIA: Lacune-small infarction	13:20	11:21	0.798
Previous infarction	No	20	9	0.023
	Small infarction (<2 cm)	11	16	
	Branch infarction	2	7	
Laboratory evaluations	HbA1c (%)	5.7 ± 0.7	6.4 ± 1.1	0.004
	Fasting glucose (mg/dl)	107.5 ± 27.5	133.8 ± 54.6	0.017
	Triglyceride (mg/dl)	120.3 ± 55.2	124.9 ± 65.7	0.760
	Total cholesterol (mg/dl)	165.5 ± 34.2	162.5 ± 46.8	0.765
	HDL-C (mg/dl)	45.1 ± 9.0	40.3 ± 12.3	0.080
	LDL-C (mg/dl)	102.9 ± 32.0	101.2 ± 40.1	0.843
	Apolipoprotein A (mg/dl)	108.8 ± 20.0	101.9 ± 26.1	0.240
	Apolipoprotein B (mg/dl)	83.8 ± 23.1	89.2 ± 29.5	0.419
	Lipoprotein(a) (mg/dl)	14.5 ± 10.4	21.9 ± 15.7	0.027
	hs-CRP (mg/l)	1.0 ± 1.0	2.1 ± 2.8	0.047
	Homocysteine (μmol/l)	9.9 ± 4.1	13.2 ± 8.9	0.063

TIA, transient ischemic attack; lacune-small infarction, lacunar or small size infarction (<2 cm-high signal on initial diffusion weighted image); NIHSS, National Institute of Health Stroke Scale; ER, emergency room; HbA1c, hemoglobin A1c; hs-CRP, high-sensitivity C-reactive protein; HDL-C, high-density lipoprotein cholesterol; LDL-C, low-density lipoprotein cholesterol

### Serum collection and miRNA extraction

Serum samples stored in the Human Bio-Resource Bank had been obtained from 3 mL of peripheral blood collected in fasting state within 24 h after admission, and had been stored at -80°C until miRNA purification. Before miRNA extraction, individual hemolysis amount was checked using a free oxyhemoglobin evaluation method;[22] only the serum samples showing <0.2 absorbance at 415 nm were selected for miRNA purification.[23]

miRNA was purified from 50 μl of serum using TaqMan^®^ miRNA ABC Purification Kit-Human-Panel A/B (part nos. 4473087 and 4473088, Applied Biosystems, Foster City, CA, USA) following the manufacturer’s protocol. The panels A and B contain superparamagnetic Dynabeads (TaqMan^®^ miRNA ABC Purification Bead Kit-HUMAN for Panel A/B, part nos. 4473085 and 4473086, Applied Biosystems) with a unique set of anti-miRNA oligonucleotides. The purified miRNAs were stored at -80°C until further experiments.

### Ethics statement

The protocol and the informed consent of the present study was approved by the Institutional Review Board of Chungnam National University Hospital (File No.: CNUH 2013-01-019). Eligible participants for the present study were informed about the purpose and experimental procedure of the study, and signed a copy of the consent form prior to participation

### miRNA microarray analysis

First, 3 μl of purified miRNA was reverse-transcribed using different primer pools (Megaplex™ RT Human Pool A [part no. 4399966] for Panel A and Pool B [part no. 4399968] for Panel B, Applied Biosystems) each containing 377 unique and 4 control miRNAs in a 10 μl final volume. The reaction mixture was thermally cycled on a PTC-0200 DNA Engine (MJ Research/Bio-Rad, Waltham, MA, USA) using the following program: 40 cycles of 2 min at 16°C, 1 min at 42°C, and 1 s at 50°C, followed by 5 min at 85°C. Then, 2.5 μl of cDNA from each pool was preamplified in a preamplification mixture (TaqMan^®^ PreAmp Master Mix, part no. 4391128) with the matching primers (Megaplex™ RT Human Pool A and B) in a final volume of 25 μl. The thermal cycles were as follows: 10 min at 95°C, 2 min at 55°C, 2 min at 72°C, 16 cycles of 15 s at 95°C and 4 min at 60°C, and a final 10 min at 99.9°C. The preamplified product was diluted in 75 μl Tris-EDTA buffer (pH 8.0) and used for microarray hybridization. The preamplified cDNA was added to two array cards containing primer sets of the primer pool A or B (TaqMan^®^ Array Human microRNA Cards A and B, Part No. 4444913, Applied Biosystems) and thermally cycled in a real-time PCR machine (QuantStudio™ 7 Flex Real-Time PCR System, Applied Biosystems) at 95°C for 10 min followed by 40 cycles of 15 s at 95°C and 1 min at 60°C.

### Selection of putative marker miRNAs from the raw microarray data

The Expression Suite software v1.0.4.4 (Applied Biosystems) was used to identify candidate atherosclerosis-predictive miRNAs from the raw microarray data. Cycle threshold (Ct) from raw microarray data—defined as the cycle at which the level of fluorescence crosses a baseline threshold—was set at 40 to capture as many expressed miRNAs as possible.[24] Undetermined values were replaced with the maximal number of cycles.[24] Before the selection of candidate marker miRNAs, we identified reference miRNAs from the 754 miRNAs included in the two microarray plates. To this end, we used previously published criteria for reference miRNAs:[25] 1) detection in all serum samples included, 2) 0.9–1.1-fold mean expression changes, and, 3) no significant expression differences (*p*>0.05) between the non-atherosclerotic and atherosclerotic patients sample groups. The stability values of the candidate miRNAs were calculated using NormFinder (http://moma.dk/normfinder-software).[26]Single or combinatorial miRNA(s) having the lowest stability value were selected as the reference miRNA(s) for the present study.

To identify candidate marker miRNAs among the 754 tested, unique miRNAs, expression differences (ΔCt = Ct _unique miRNA_-Ct _reference miRNA(s)_) among the individual unique miRNAs were evaluated in each patient. Then, the relative quantity of expression (Q_rel_ = 2^−ΔCt^) of each unique miRNA was calculated.[25] miRNAs that showed <0.5- or >2.0-fold-change differences in the Q_rel_ value between the two groups with *p* < 0.05 were selected as candidate marker miRNAs. Then, we carried out discriminant analysis with the candidate miRNAs to select miRNAs related to atherosclerosis presence (candidate miRNAs with *p* < 0.05 in the discriminant analysis), which were used to evaluate expression differences between the non-atherosclerotic and atherosclerotic groups. The data obtained by the microarray are freely available in the NCBI Gene Expression Omnibus repository with the accession number GSE96621.

### Validation of expression differences of the putative marker miRNAs between non-atherosclerotic and atherosclerotic groups

We used real-time RT-PCR to validate the expression differences of the putative marker miRNAs between the non-atherosclerotic and atherosclerotic groups, using serum collected from 33 non-atherosclerotic and 32 atherosclerotic patients. Three microliters of purified miRNA were reverse-transcribed with customized primer pairs for the putative marker and two reference miRNAs (miR-212, -372, -474, and -744 as putative marker miRNAs; miR-197 and -374 as reference miRNAs) in a 10-μl reaction mixture. The thermal cycles were as follows: 40 cycles of 2 min at 16°C, 1 min at 42°C, and 1 s at 50°C, followed by a final 5 min at 85°C. Then, 2.5 μl of cDNA was preamplified in a pre-amplification mixture (TaqMan^®^ PreAmp Master Mix, part no. 4391128) with the matching primers in a 25-μl reaction volume. The pre-amplification mixture was thermally cycled for 10 min at 95°C, 2 min at 55°C, 2 min at 72°C, followed by 16 cycles of 15 s at 95°C, 4 min at 60°C, and a final incubation of 10 min at 99.9°C. The pre-amplified product was diluted with 75 μl in Tris-EDTA buffer (pH 8.0). Finally, the target and reference miRNAs were individually subjected to real-time PCR with corresponding primer sets in a 20-μl PCR mixture (TaqMan^®^ Universal Master Mix II, No AmpErase^®^ UNG, Part No. 4324018, Applied Biosystems) on a Plus-One thermal cycler (Applied Biosystems) using the following cycles: 10 min at 95°C, followed by 40 cycles of 15 s at 95°C and 1 min at 60°C. The difference of Q_rel_ for each putative marker miRNA, which was calculated based on the expression differences (Δ_Ct_) between the individual putative marker miRNA and the reference miRNAs (miR-197 and -374), was compared between non-atherosclerotic and atherosclerotic groups. (S1 Table)

### Statistical analysis

Differences in sex and histories of 4 risk factors (hypertension, diabetes, smoking, and alcohol drinking) were compared using a chi-squared test. Differences in age, body mass index, and 10 laboratory markers for cardiovascular risk (HbA1c, hs-CRP, triglyceride, total cholesterol, HDL-C, LDL-C, apolipoprotein A and -B, lipoprotein(a), and homocysteine), incidence of previous ischemic stroke lesion on MRI and expression of 4 target miRNAs (miR-212, -372,-454, and -744) were compared using Student’s *t*-test. Associations of the clinical and laboratory cardiovascular risk factors and the 4 identified miRNAs with the presence of atherosclerosis were tested by forward logistic regression analysis (Table 1).

Overfitting of the logistic regression model was evaluated using a leave-one-out cross-validation analysis conducted in 65 patients. The accuracy of the model was assessed using sensitivity and specificity analysis of the factors included in the logistic model. The discriminative power of the significant factors in the logistic regression model was evaluated by receiver operating characteristic curve analysis. Individual and combinational areas under receiver operating characteristic curve (AUC) of the risk markers and/or miRNAs included in the model were compared between the non-atherosclerotic and atherosclerotic groups. Net reclassification improvement (NRI) and integrated discrimination improvement (IDI) were calculated to evaluate whether the validated miRNAs improved reclassification of the atherosclerosis presence estimated with the cardiovascular risk markers included in the logistic model.[27] NRI was calculated using the following formula: $\hat{NRI}=\frac{\sum_{i}v(i)}{\text{No}.\;\text{of}\;\text{events}}-\frac{\sum_{j}v(j)}{\text{No}.\;\text{of}\;\text{nonevents}}$, where No. of events is the number of patients classified as atherosclerotic, No. of nonevents is the number of patients classified as non-atherosclerotic, *v(i)* is a movement indicator: any increase in predicted probabilities for individuals with events means upward movement (*v(i)* = 1), any decrease is a downward movement (*v(i)* = -1), and no change is no movement (*v(i)* = 0). IDI was calculated using the following formula: $\hat{IDI}=({\bar{\hat{p}}}_{new-events}-{\bar{\hat{p}}}_{old-events})-({\bar{\hat{p}}}_{new-nonevents}-{\bar{\hat{p}}}_{old-nonevents})$, where ${\bar{\hat{p}}}_{new-events}$ is the mean of the new model-based predicted probabilities of an event (atherosclerotic) for those who develop events, ${\bar{\hat{p}}}_{old-events}$, the corresponding quantity based on the old model, ${\bar{\hat{p}}}_{new-nonevents}$, the mean of the new model-based predicted probabilities of an event for those who do not develop events, and ${\bar{\hat{p}}}_{old-nonevents}$, the corresponding quantity based on the old model. All statistical analyses were performed using SPSS ver. 22.0 (SPSS Inc., Chicago, IL, USA) and SAS ver. 9.3 (SAS Institute Inc., Cary, NC, USA).

## Results

### Patient selection and clinical and laboratory cardiovascular risk factor evaluation

Initially, 73 patients (non-atherosclerotic, 37; atherosclerotic, 36) were selected for the study based on the presence of severe stenosis (Fig 1). After review of the clinical and laboratory risk factors and raw microarray data, 8 patients were excluded because of missing laboratory data for at least one tested parameter (4 patients), unsuccessful microarray assay (1 patient), or insufficient serum sample (3 patients). Finally, 65 patients (non-atherosclerotic, 33; atherosclerotic, 32) were included in the present study.

On clinical severity evaluations performed using NIHSS, both atherosclerotic and non-atherosclerotic group was similar at ER and at discharge in both groups without statistical significance. (Table 1) With regard to clinical risk factors, no significant differences were observed in age, sex ratio, and body mass index between non-atherosclerotic and atherosclerotic patients (Table 1). With regard to laboratory risk factors, atherosclerotic patients had higher HbA1c, hs-CRP, lipoprotein(a), fasting glucose, and homocysteine. Instead, HDL-C tended to be lower in atherosclerotic than non-atherosclerotic patients. Triglyceride, total cholesterol, LDL-C, apolipoprotein-A/B were not significantly different between the two groups (Table 1). On the MRI review for previous lesions, no subacute lesion was observed in both atherosclerotic and no-atherosclerotic groups. Instead, old small infarctions (Atherosclerotic, 16; No-atherosclerotic group, 11 patients) and old branch infarction (Atherosclerotic, 7; No-atherosclerotic group, 2 patients) were more frequent on Atherosclerotic then No-atherosclerotic group. (Table 1)

### Identification of putative atherosclerosis-related marker miRNAs by microarray expression profiling

miRNA microarray profiling was initially conducted using serum samples of 8 out of the 37 non-atherosclerotic and 8 out of the 36 atherosclerotic patients who had been selected for the study based on the presence of severe stenosis. Microarray data from 1 patient were excluded due to unsatisfactory fluorescence; thus, raw microarray data from 8 non-atherosclerotic and 7 atherosclerotic patients were analyzed.

We selected 25 miRNAs as candidates for reference miRNA based on the criteria for reference miRNAs (Table 2). After NormFinder analysis of the 25 miRNAs, the pair miR-197/-374 showed the lowest stability value (Table 2). Therefore, we used this pair as reference miRNAs to obtain individual expression levels (Δ_Ct_) of all unique miRNAs included in the microarray panels. Then, relative quantity of expression (Q_rel_) values of individual unique miRNAs were calculated using the individual Δ_Ct_.

**Table 2:** Stability values of candidate reference miRNAs calculated with NormFinder.

Gene name	Stability value
let-7c	0.26
miR-126#	0.176
miR-1274B	0.207
miR-1305	0.162
miR-136	0.397
miR-145	0.303
miR-151-3p	0.177
miR-155	0.249
miR-185	0.368
miR-191	0.135
miR-192	0.186
miR-197	0.098
miR-19a	0.149
miR-19b	0.142
miR-202	0.247
miR-20a	0.167
miR-30a-3p	0.283
miR-340	0.336
miR-345	0.387
miR-374	0.102
miR-375	0.348
miR-422a	0.359
miR-425-5p	0.151
miR-573	0.32
miR-645	0.33
miR-197/miR-374	0.073

**Fig 2:**
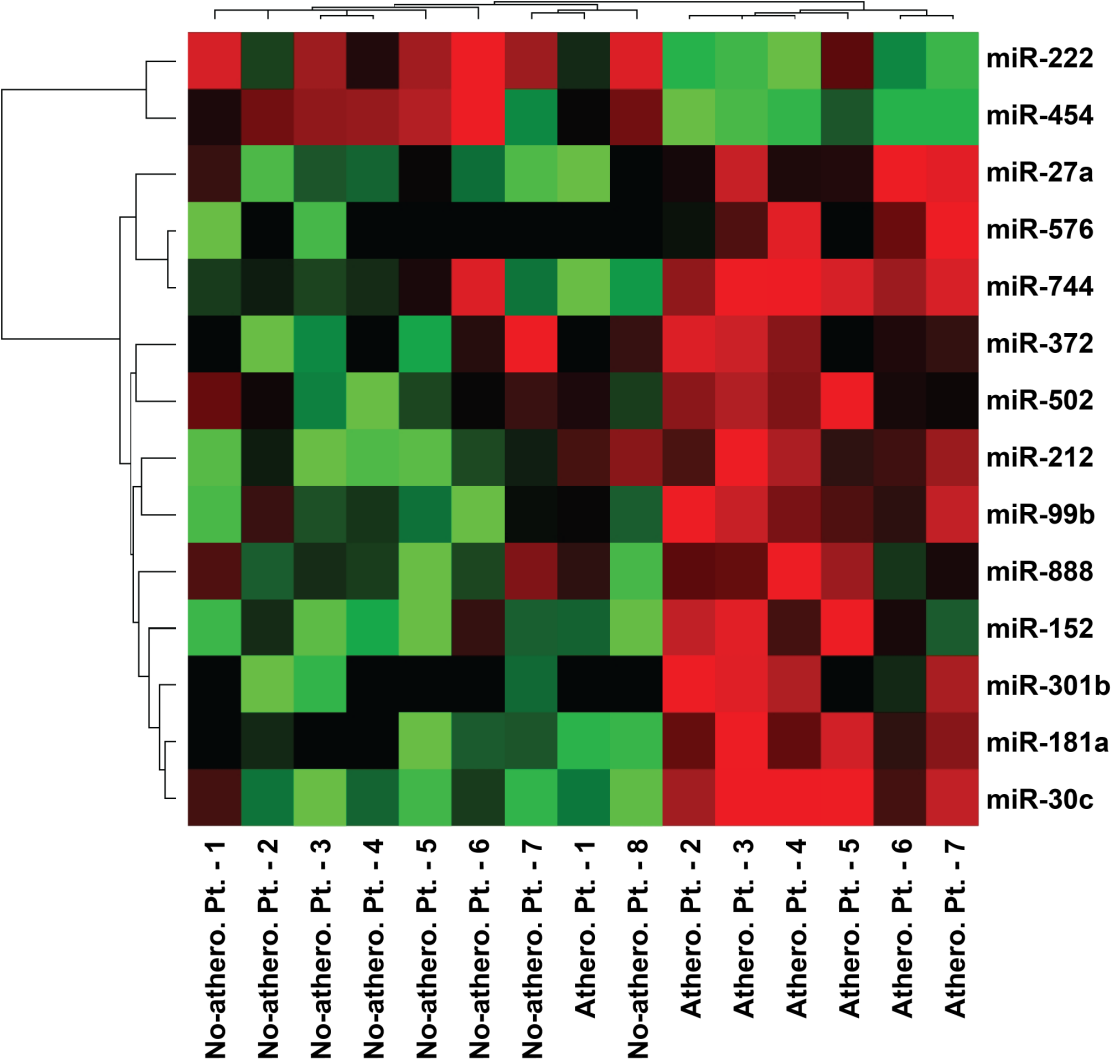
Heat map of the 14 differentially expressed candidate microRNAs obtained from non-atherosclerotic (no-athero. pt.) and atherosclerotic patients (athero. pt.).

Upon evaluation of Q_rel_, 14 miRNAs (miR-30c, -99b, -152, -181, -212, -222, -301b, -372, -454, -502, -576, -27a, -744, and -888) showed differential expression between non-atherosclerotic and atherosclerotic patients (Table 3, Fig 2). To determine most valid ones of 14 miRNAs to statistically estimate atherosclerosis presence, we performed a stepwise discriminant analysis. Of these, miR-454 (F = 22.62), miR-744 (F = 16.13), miR-372 (F = 6.57), and miR-212 (F = 5.07) showed *p* < 0.05 in the discriminant analysis, and thus, were selected as marker miRNAs for the validation study.

**Table 3:** Candidate miRNAs showing expression differences between non-atherosclerotic and atherosclerotic patient groups.

miRNA	Expression difference analysis (Q_rel_^*^, mean ± SD)		
	Non-atherosclerotic	Atherosclerotic	*p*-value
miR-181a	0.0855 ± 0.0241	0.3400 ± 0.0579	0.0009
miR-454	0.7692 ± 0.1151	0.1765 ± 0.0163	0.0012
miR-30c	1.9176 ± 0.2679	4.8680 ± 0.6935	0.0044
miR-99b	0.1474 ± 0.0336	0.5282 ± 0.1005	0.0082
miR-744	0.1124 ± 0.0515	0.3763 ± 0.0697	0.0085
miR-212	0.2505 ± 0.0637	0.5536 ± 0.0916	0.0159
miR-222	15.1756 ± 2.3675	6.5891 ± 2.0868	0.0188
miR-372	0.0032 ± 0.0018	0.0219 ± 0.0060	0.0203
miR-576	0.0009 ± 0.0005	0.0154 ± 0.0048	0.0231
miR-502	0.1998 ± 0.0558	0.5967 ± 0.1361	0.0272
miR-301b	0.0003 ± 0.0001	0.0268 ± 0.0093	0.0292
miR-888	103.8126 ± 29.0517	350.7029 ± 92.5094	0.0375
miR-27a	0.1789 ± 0.0443	0.5827 ± 0.1529	0.0388
miR-152	0.0684 ± 0.0138	0.2617 ± 0.0751	0.0421

^*^Q_rel_ = 2^−ΔCt^

### Validation of 4 marker miRNAs in atherosclerotic patients

Real-time RT-PCR analysis of the 4 marker miRNAs (miR-212, -372, -454, and -744) was used to validate the expression differences between non-atherosclerotic (n = 33) and atherosclerotic (n = 32) patients. Of these 4, only miR-212 showed a significantly higher Q_rel_ in atherosclerotic than in non-atherosclerotic patients (Table 4). Analysis of the relationships between miR-212 expression and disease classification, TIA, or lacunar-small infarctions, revealed that the Q_rel_ of miR-212 was not significantly correlated with the presence of lacunar-small cerebral infarction (TIA vs. lacunar-small infarction patients: 0.038 ± 0.030 vs. 0.047 ± 0.050, *p* = 0.407) (Table 1). On the comparison of expression differences by the presence of the previous cerebral infarctions, the expression of all four miRNAs (miR-212, No: 0.039±0.041, Small: 0.044±0.041, Branch infarctions: 0.055±0.062, *p* = 0.668; miR-372, No: 0.001±0.002, Small: 0.002±0.003, Branch infarctions: 0.001±0.002, *p* = 0.788; miR-454, No: 1.579±5.551, Small: 1.145±2.687, Branch infarctions: 0.461±0.766, *p* = 0.789; miR-744, No: 0.019±0.035, Small: 0.041±0.109, Branch infarctions: 0.011±0.014, *p* = 0.463) was not different by the presence of chronic infarctions observed in initial MRI images.

**Table 4:** Expression differences of the 4 target miRNAs between non-atherosclerotic and atherosclerotic patients.

Name of miRNA	Expression changes (Q_rel_^*^)		*p*-value
	Non-atherosclerotic (mean ± SD, n = 33)	Atherosclerotic (mean ± SD) (n = 32)	
miR-212	0.027 ± 0.026	0.062 ± 0.062	0.003
miR-372	0.002 ± 0.003	0.001 ± 0.002	0.653
miR-454	1.214 ± 2.532	1.315 ± 2.895	0.882
miR-744	0.023 ± 0.039	0.077 ± 0.156	0.068

^*^Q_rel_ = 2^−ΔCt^

### Interaction of miR-212 with cardiovascular risk factors

To evaluate whether the 4 marker miRNAs independently related with atherosclerosis presence assessed with the other cardiovascular markers, we performed logistic regression analysis of the 4 miRNAs (miR-212, -372, -454, and -744), and all clinical (age, body mass index, and history of hypertension) and laboratory (HbA1c, fasting glucose, triglyceride, total cholesterol, HDL-C, LDL-C, apolipoprotein A and B, lipoprotein(a), hs-CRP, and homocysteine) cardiovascular risk markers tested in the present study (Table 1). miR-212 and HbA1c, HDL-C, and lipoprotein(a) were identified as independent variables associated with atherosclerosis presence (Table 5). Leave-one-out cross validation to evaluate overfitting of the regression model revealed that the accuracy of atherosclerosis-presence estimation by HbA1c, HDL-C, and lipoprotein(a) was 73.8%. Inclusion of miR-212 in the model increased the risk estimation to 76.9%.

**Table 5:** Multivariate analysis using 4 target miRNAs and markers for cardiovascular risk factors.

Variables	Multivariate analysis			
	Exp(B)	95% CI		*p*-value
		Lower limit	Upper limit	
Constant	0.003	–	–	0.057
HbA1c	2.929	1.327	6.467	0.008
HDL-C	0.934	0.87	1.003	0.059
Lipoprotein(a)	1.065	1.01	1.123	0.02
miR-212	2.601.E+11	32.231	2.098.E+21	0.024

HbA1c, hemoglobin A1c; HDL-C, high-density lipoprotein cholesterol

### Accuracy and discriminatory power increment of miR-212

The accuracy of HbA1c for the estimation of atherosclerosis presence was 70.8%. In combination with HDL-C and lipoprotein(a), the accuracy increased to 78.5%. Upon addition of miR-212 to the risk estimation model, the accuracy was increased to 84.6% (Table 6). The AUC of individual variables were modest (range, 0.6780–0.7491). Combination of the 3 risk factors increased the AUC to 0.8258. Upon addition of miR-212, the AUC increased to 0.8646 (Table 6).

**Table 6:** Accuracy and discrimination analysis using individual and combined markers.

Variables	Performance measurements			AUC
	Specificity	Sensitivity	Accuracy	
HbA1c	75.8	65.6	70.8	0.7491
HDL-C	51.5	68.8	60.0	0.6690
Lipoprotein(a)	75.8	46.9	61.5	0.6780
miR-212	81.8	46.9	64.6	0.6866
HbA1c, lipoprotein(a), HDL-C	81.8	75.0	78.5	0.8258
HbA1c, lipoprotein(a), HDL-C, miR-212	87.9	81.3	84.6	0.8646

HbA1c, hemoglobin A1c; HDL-C, high-density lipoprotein cholesterol; AUC, areas under the receiver operating characteristic curve

### Reclassification of atherosclerosis presence by inclusion of miR-212 in the risk model

In the reclassification analysis, when HbA1c, HDL-C, and lipoprotein(a) were included in the estimation model, the mean number of misclassified patients was 9 out of 32 patients in the atherosclerotic group and 8 out of 33 patients in the non-atherosclerotic group. However, when miR-212 was added, 3 (33.3%) of 9 misclassified patients in the atherosclerotic group and 2 (25%) of 8 misclassified patients in non-atherosclerotic group were reclassified. However, 1 (4.3%) of 23 properly classified patients in the atherosclerotic group and 2 (8%) of 25 properly classified patients in the non-atherosclerotic group were misclassified after the addition of miR-212. Overall, reclassification of atherosclerosis presence was improved by the addition of miR-212 to the three standard risk markers in both NRI ($\hat{NRI}$ = 0.4527, *p* = 0.034) and IDI ($\hat{IDI}$ = 0.0737, *p* = 0.047) analysis.

## Discussion

The present study identified miR-212 as a novel marker that enhanced the estimation power of three established cardiovascular risk markers, HbA1c, HDL-C, and lipoprotein(a), for atherosclerosis presence in ischemic stroke patients. Several miRNAs have been already reported to be related to atherosclerosis or cardiovascular diseases.[9,28,29] However, interactive effects of these miRNAs with standard and/or established cardiovascular risk factors in the estimation of atherosclerosis presence were not known.

We evaluated the individual and synergistic significance of miR-212 as a novel atherosclerosis estimation marker by following the recommendations of a previous protocol for novel cardiovascular markers.^3^ Our findings corroborated the usefulness of a rigorous study design with strict inclusion criteria stratified by factors influencing circulating miRNAs. Previous studies identified influences of age,[30] lesion size of ischemic stroke[31], and, time lapse from symptom onset to blood sampling [32] on the expression of circulating miRNAs. Thus, to lower the influences of age of patient and lesion size, we included TIA or lacunar-small infarction (<2-cm lesion size on initial DWI) patients, who were >65 years old. And, to lower the influences of time lapse from symptom onset to blood sampling, the blood samples were obtained within 24 h after admission and within 48 h after symptom onset from all included patients.

We used cardiovascular risk markers of dyslipidemia, diabetes, inflammation, and endothelial dysfunction that have been used in the Atherosclerosis Risk in Communities[33] and Framingham Heart[34] studies (Table 1). Among the 16 cardiovascular risk markers included in the present study, only HbA1c, HDL-C, and lipoprotein(a) showed significant correlation with atherosclerosis. Individual relationships between these three markers and atherosclerosis have been described in previous studies.[35–37] As indicated by analysis of the accuracy and discriminatory power, the estimation of atherosclerosis presence was improved by combining the three markers, even though the individual estimation role of the markers was modest.

Microarray experiments usually involve numbers of genes that largely exceed the number of cases to be studied.[38] Thus, even when multiple candidate genes are initially obtained as putative markers related with specific diseases after raw microarray data analysis,[39] just a few genes might be statistically valid as candidate genes.[38,40] To select potential marker miRNA subsets from the initial candidate miRNAs, filtering and pre-selection using discriminant functions[38,41] or the co-inertia approach[42] have been applied. In the present analysis, we initially identified 14 candidate miRNAs showing differential expression between the non-atherosclerotic and atherosclerotic groups from a total of 754 miRNAs. After discriminant analysis of the initial 14 candidates to determine putative marker miRNAs, miR-212, -372, -454, and -744 were selected for the validation study. miR-212 was the only miRNA showing a significantly different expression between non-atherosclerotic and atherosclerotic patients in the validation experiment, and was included in following accuracy and discriminatory power analysis with the three circulating risk markers, HbA1c, HDL-C, and lipoprotein(a).

miR-212 synergistically improved the estimating power of the combined 3 cardiovascular risk markers. Logistic regression analysis in a validation population revealed that all 4 tested factors were independent, predictive factors. Accuracy and discrimination analysis based on AUC measurement showed that miR-212 synergistically improved the estimating power of the combined three circulating risk markers. Even though the *c* statistic shows the probability that the measure or predicted risk is higher for an atherosclerotic patient than for a non-atherosclerotic patient,[43] it is not informative of the probability of the predictive value.[44] Thus, we performed a reclassification analysis using NRI and IDI to assess whether a miR-212 also improved the reclassification power of the three markers. Indeed, in both NRI and IDI analysis, reclassification was also improved by the addition miR-212 to the estimation model. Thus, the discrimination and reclassification analyses showed the robustness of miR-212 as a novel marker for enhanced estimation of atherosclerosis presence in combination with HbA1c, HDL-C, and lipoprotein(a).

The promising result of miR-212 as a novel maker for atherosclerosis warrants future studies to evaluate functions of miR-212 in atherosclerosis development and to assess its clinical value in patient management and outcome evaluations.[3] Functional evaluation should include both miR-212 and miR-132 because both miRNAs share the same seed sequences and are located very closely on chromosome 17p13.3.[45] Expression changes of miR-212/-132 have been reported in neuronal development and degeneration as well as in various diseases including different cancers and in drug addiction.[45] Interestingly, miR-212/-132 expression changes have been reported in inhibition of endothelial migration [46] and in risk factors of atherosclerosis such as glucose metabolism,[47,48] hypertension,[49] and inflammation.[50] However, a direct relationship of miR-212 with atherosclerosis had not been established to date. The present identification and validation of miR-212 in atherosclerosis patients provides strong evidence of a relationship between miR-212 and atherosclerosis, even though basal level and environmental influences of miR-212 expression has not been well known, yet. Future studies will have to evaluate the relationships between miR-212 and atherosclerosis development in *in vivo* model and humans.

The present study provided proof-of-concept for the use of miR-212 as a novel marker to enhance estimation of atherosclerosis presence by HbA1c, HDL-C, and lipoprotein(a) in ischemic stroke patients in whom atherosclerosis severity was classified in an “all-or-nothing” fashion. However, most patients showed variable severity of atherosclerosis on MRA and/or carotid duplex examinations; therefore, prospective cohort studies [3] in patients classified by atherosclerosis severity based on well-designed criteria are needed.

## Conclusions

The present study identified and validated circulating miR-212 as a marker of atherosclerosis. In particular, synergy of miR-212 with the three standard cardiovascular risk markers, HbA1c, HDL-C, and lipoprotein(a), was verified with regard to accuracy, discrimination, and reclassification by following a protocol recommended for the evaluation of novel markers of cardiovascular risk.[3] Even though more prospective studies for the use of miR-212 as a novel marker to predict atherosclerosis in clinical practice are needed, the present data showed that miR-212 is a useful circulating marker to individually and synergistically improve the estimation of atherosclerosis presence.

## Supporting information

S1 Table Clinical and microRNA expression data of all included patients. (XLSX)
