# Correction: Synergy of circulating miR-212 with markers for cardiovascular risks to enhance estimation of atherosclerosis presence

**DOI:** 10.1371/journal.pone.0184600

**Published:** 2017-09-06

**Authors:** The PLOS ONE Staff

The following information is missing from the Funding section: This study was supported by the Basic Science Research Program through the National Research Foundation of Korea funded by the Ministry of Education grant 2016R1A6A3A11933930 to JYK. This study was supported by the Chungnam National University Hospital Research Fund 2016 of the Chungnam National University Hospital to HSJ. The publisher apologizes for the errors.
